# Towards Enhancement of Performance of K-Means Clustering Using Nature-Inspired Optimization Algorithms

**DOI:** 10.1155/2014/564829

**Published:** 2014-08-18

**Authors:** Simon Fong, Suash Deb, Xin-She Yang, Yan Zhuang

**Affiliations:** ^1^Department of Computer and Information Science, University of Macau, Macau; ^2^Department of Computer Science and Engineering, Cambridge Institute of Technology, Ranchi 835103, India; ^3^School of Science and Technology, Middlesex University, The Burroughs, London NW4 4BT, UK

## Abstract

Traditional K-means clustering algorithms have the drawback of getting stuck at local optima that depend on the random values of initial centroids. Optimization algorithms have their advantages in guiding iterative computation to search for global optima while avoiding local optima. The algorithms help speed up the clustering process by converging into a global optimum early with multiple search agents in action. Inspired by nature, some contemporary optimization algorithms which include Ant, Bat, Cuckoo, Firefly, and Wolf search algorithms mimic the swarming behavior allowing them to cooperatively steer towards an optimal objective within a reasonable time. It is known that these so-called nature-inspired optimization algorithms have their own characteristics as well as pros and cons in different applications. When these algorithms are combined with K-means clustering mechanism for the sake of enhancing its clustering quality by avoiding local optima and finding global optima, the new hybrids are anticipated to produce unprecedented performance. In this paper, we report the results of our evaluation experiments on the integration of nature-inspired optimization methods into K-means algorithms. In addition to the standard evaluation metrics in evaluating clustering quality, the extended K-means algorithms that are empowered by nature-inspired optimization methods are applied on image segmentation as a case study of application scenario.

## 1. Introduction

**Figure 1:**
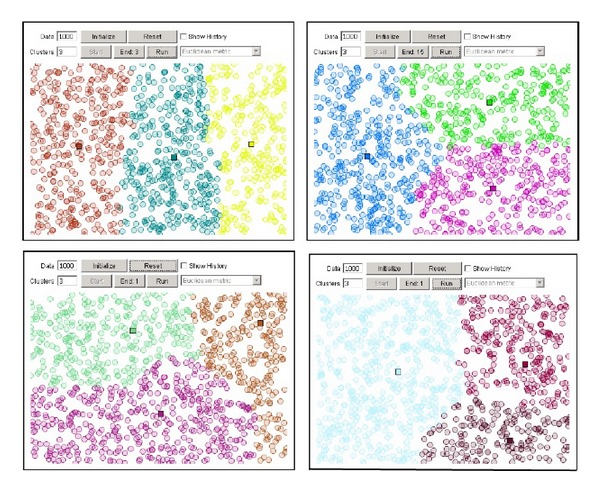
Examples of clustering results by random centroids of K-means where *k* = 3.

Based on a partitioning strategy, K-means clustering algorithm [1] assigns membership to data points by measuring the distance between each pair of data point and centroid of a designated cluster. The membership assignment will be progressively refined until the best possible assignment is yielded—that is when the total intradistances of the data points within a cluster are minimized and the total interdistances of the data points across different clusters are maximized. The final quality of the clustering results, however, depends largely on the values of the initial centroids at the beginning of the partitioning process. These initial centroid values are randomly generated each time the clustering kick-starts which are different from time to time. By such random chance, K-means can probably plunge into local optima whereby the final quality of the clusters falls short from the globally best. An example that is depicted in Figure 1 demonstrates some possible outcomes of K-means. The top two snapshots represent good clustering results where the cluster distributions are even; the bottom two snapshots show otherwise, the clustering results in uneven distribution. All these depend on the starting positions of the centroids which are randomly generated.

Technically it is possible though not feasible in achieving a globally optimum clustering result, via a brute-force approach in trying out exhaustively all partitioning possibilities. As the number of clusters and the number of data points increase, the combinatorial number of possible grouping arrangements escalates, leading to computationally prohibitive. Therefore, heuristic approach is desired for seeking global optima stochastically, improving the quality of the final clustering results iteration by iteration. Metaheuristics which enable incremental optimization by design are ideal candidates for such computation.

A substantial collection of nature-inspired optimization methods aka metaheuristics have emerged recently with designs mimicking swarm behavior exhibited by living creatures. Each of the search agents represents a particular combination of centroid positions; they move and search for optimality in their own ways, they sometimes communicate with each other and are collectively guided towards a global optimization goal. To date, the proposed nature-inspired optimization algorithms have gained much attention among data mining researchers. The computational merits have been verified mathematically and their feasibilities have been applied in various practical applications. However, validating the efficacy of hybrids combining such nature-inspired algorithms with classical data mining algorithms is still at an infant stage [2].

By the merits of design, nature-inspired optimization algorithms are believed to be able to overcome the shortcomings of K-means clustering algorithms on the issue of getting stuck in local optima. The objective of this study is to validate the efficacy of the hybrids and to quantitatively measure the quality of clustering results produced by each of the hybrids. In our experiments, we used four popular nature-inspired optimization methods to combine with K-means clustering algorithm. The integration is paramount, because it enables enhancement over data mining algorithms which have many wide applications.

A preliminary experiment reported in [3] shows their feasibility as a successful pioneer exemplar. This paper reports further experiment including a case study of image segmentation using these hybrid algorithms. In the past, some researchers started to integrate nature-inspired optimization methods into K-means algorithms [4]; their research efforts are limited to almost the same form of swarming maneuvers—that is, there is constantly a leader in the swarm which the fellow agents follow. Some examples are nature-inspired optimization algorithms such as the Artificial Bee Colony [5], Firefly [6], and Particle Swarm Optimization [7]. For the sake of intellectual curiosity, in our experiments as well as whose models described in this paper, two nature-inspired algorithms which take on a slightly different course of swarming are included. They are the Bat Algorithm [8] which swarm with varying speeds and the Cuckoo Algorithm [9] which do not swarm but iterate with fitness selection improvement. They represent another two main groups of algorithms and their variants, which are adaptive to the environment by their sensing abilities, and utilize a special method to improve the solution by evolving the old solutions from one generation into better ones in new generations. Specifically, the performance indicators in terms of speed and time consumption for clustering by these two bioinspired algorithms integrated with K-means are observed. The technical details of the aforementioned nature-inspired algorithms and the K-means clustering are not duplicated here. Readers are referred to the respective references for the background information of the algorithms involved in this paper.

## 2. Enhancing K-Means Clustering by Nature-Inspired Optimization Algorithms

A major reason in obtaining quality K-means clustering results is having the right combination of centroid positions. The resultant centroids ideally should be dispersed in such a way that the clusters formed upon them yield the maximum quality, which we call a global optimum. It is characterized by having the properties of maximum intrasimilarities and minimum intersimilarities in clustering. K-means is known to produce clustered data with nonoverlapping convex clusters, and they always converge quickly. One drawback is that clusters in K-means do not always converge into the global optimum. Just like any other partition-based clustering algorithm, initial partition is made by some randomly generated centroids which do not guarantee the subsequent convergence will lead to the best possible clustering result. K-means, for example, is reputed to have the final clusters stuck at local optima which prevent further exploration for the best results [10]. In reality, to achieve the best clustering results, it would require running many rounds with each round taken different random initiations of centroid values. This practice certainly would have to be done at the very high cost of computation and model training time. In [3], the authors first proposed the integration of nature-inspired optimization algorithms into K-means. We take a step further in the experiments and applying it for image segmentation. To start with the integration, the formation of centroids, which are computed stochastically from start to convergence, is directed by the searching agents of the nature-inspired optimization algorithms. The evolution of the new generation is based on the principle that the centroids which are being relocated in each iteration are inclined to enable the new clusters that are being formed with better results. Hence, in order to achieve the optimal configuration of centroids as an objective, we let cen_*jv*_ be the centroid as the center point of the *j*th cluster in the multidimensional search space by the *v*th attribute. *W*_*i*,*j*_ is the membership of data point *x*_*i*_ whether it exists in cluster *j*. The centroid location can be calculated by (2) for each attribute *v* and for each cluster *j*; the clustering objective function is defined as (3):

(1) $$\begin{array}{c} w_{i,j}=\{\begin{array}{cc} 1, & x_{i}\in {\text{cluster}}_{j},\\ 0, & x_{i}\notin {\text{cluster}}_{j}, \end{array} \end{array}$$

(2) $$\begin{array}{c} {\text{cen}}_{j,v}=\frac{\sum_{i=1}^{S}w_{i,j}x_{i,v}}{\sum_{i=1}^{S}w_{i,j}},\;j=1,\ldots ,K,\;\;v=1,\ldots ,K*D, \end{array}$$

where *S* is the number of search agents in the whole population, *K* is the maximum number of clusters, and *j* is the current cluster being processed. The highest dimension of attributes is *D* for the dataset; a centroid is hence located by a tuple of size *D*. In the design of our computational model, cen is a 2D matrix of size *K* × *D* holding all the values of the centroids (the cluster centers) indicated by cen_*j*,*v*_:

(3) $$\begin{array}{c} F\left(\text{cen}\right)=\overset{K}{\underset{j=1}{\sum }}\;\overset{S}{\underset{i=1}{\sum }}w_{i,j}\overset{K*D}{\underset{v=1}{\sum }}{\left(x_{i,v}-{\text{cen}}_{j,v}\right)}^{2}. \end{array}$$

The computation process scans through the cen matrix up to *K* × *D* times to check the values of all the attributes of the data point *x* for measuring the distance or similarity between each pair of *x* and the centroid. This process repeats for each cluster *v*. For the optimization algorithm to work, each searching agent represents a particular combination of centroids for all the clusters, as an optimization solution in the *K* × *D* dimensional search space. The best search agent being found in each iteration is supposed to produce the best clustering result in that particular iteration. For instance, in a simple dual-cluster clustering task, there are three variables for the objective function to work with. In this case, there are three dimensions in the search space. In the three dimensional search space, the *i*th search agent may take the form of *x*_*i*_ = (*i*, [*x*_*i*,1_, *x*_*i*,2_, *x*_*i*,3_, *x*_*i*,4_, *x*_*i*,5_, *x*_*i*,6_]). Due to the fact that there are 2 × 3 attributes for a search agent, the centroid can be coded in the same format for the coordinate of the second dimension. A search agent may have a best fitness value as cen = (*x*_*i*,1_, *x*_*i*,2_, *x*_*i*,3_, *x*_*i*,4_, *x*_*i*,5_, *x*_*i*,6_). According to the given definitions, the clustering strategy can be constructed as a minimization function, as follows, that aims at shortening the distances among the data points in a cluster:

(4) $$\begin{array}{c} {\text{clmat}}_{i,j}=\underset{k\in K}{\min}\left\{||x_{i}-{\text{cen}}_{k}||\right\}. \end{array}$$

The ranges of the parameters are as follows: *i* = 1,…, *N*, *j* = 1,…, *S*, and *k* = 1,…, *K*. The double line notation in (4) means it is a function of Euclidean distance. The interpretation of (4) is that the *i*th search agent that is now handling the *k*th cluster takes a value by measuring the minimized distance between the *i*th search agent and centroid of the *k*th center. The equation is an objective function in which the smaller the value the better. As long as the value of clmat is minimized by this metaheuristic optimization approach, every data point within a cluster would be drawn as close as possible to the centroid. The metaheuristic will guide the search agents to find the appropriate centroids for the clusters.

Nature-inspired optimization algorithms require certain functional parameters to be initiated with values to run. The function parameters are defined as follows. They allow the users to set with user-specific values for customizing the operations of the algorithms. Some of the parameters are common across different bioinspired optimization algorithms. In this paper, we have four hybrids, which resulted from combining four bioinspired optimization algorithms into K-means. With the capital letter C denoting “clustering,” the four hybrid algorithms are called C-ACO, C-Bat, C-Cuckoo, and C-Firefly, respectively. The original mathematical models for the four bioinspired optimization algorithms can be found in [6, 8, 9, 11], respectively.

Table 1 consists of the parameters for the C-Firefly algorithm. *X* is a composite matrix of size [*N*, (*K* ⊗ *D*)], where *x* ∈ *X* since *X* has a maximum of *K* centroids and each centroid is represented by a maximum of *D* dimensions by the attributes.

The C-Bat algorithm has more parameters than the others because it includes the velocity and location of each bat (Table 3). Velocity is determined by frequency, loudness, and pulse rate. However, only two of the four bioinspired clustering algorithms (C-Cuckoo and C-Bat) are described in this paper due to space limitations. Nonetheless, C-Firefly and C-Bat have recently been reported in [1]. Readers may refer to [1] for the detailed integration of a K-Means clustering algorithm with the firefly and bat algorithms.

### 2.1. Cuckoo Clustering Algorithm (C-Cuckoo)

In the original cuckoo algorithm, Yang and Deb used an analogy whereby each egg in a nest represents a solution, and a cuckoo egg represents a new solution. The goal is to use the new and better solution to replace a relatively poor solution by chasing off an old egg with a new cuckoo egg. We adopt the same analogy in constructing our C-Cuckoo algorithm. The solution *x* represents the host nest. In the clustering algorithm, the solution is composed of a set of real numbers representing the cluster center. As defined earlier, *x* takes the form of a (*N*, *K* ⊗ *D*) matrix where *N* is the population, *K* is the number of clusters, and *D* is the number of attributes associated with each data point. The second index of the matrix represents the center of all *K* clusters, and the whole *x* represents the current locations of all the cuckoos. We now give a simple example. If there is a given data set and two dimensions and we need to create two clusters (*K* = 2, *D* = 2), then the value of *x* is four. The *i*th *x* in the middle of the clustering process may look something like this:

 Cluster 1: 1 3
 Cluster 2: 8 9

In the initialization phase, the population of *n* host nests *x*_*i*_, where *i* = 1,2,…, *n*, is generated. The cluster centers are represented by the means of the attributes. Each cuckoo has the same parameters (Table 2):* Tol* (tolerance) and* pa* (alien egg discovery rate). In this phase, the most important action is that a cluster ID is randomly assigned to each cuckoo as the initial clustering result.

Because the cuckoo has characteristics typical of Levy flight, when it comes to generating a solution *x* ^(*t*+1)^ for cuckoo *i*, we use the equation *x* ^(*t*+1)^ = *x* ^(*t*)^ + *δ* ⊕ Levy(*λ*), where *δ* is the step size scalar used to control the resolution of the step length. In our algorithm, we use *δ* = 1, which satisfies most cases. The above formula means the cuckoo takes a random walk. In this case, the random walk is implemented as a Levy flight, which is based on Mantegna's algorithm [12]. The algorithm takes the following steps. First, the number of centroids *k* is initialized, as are the other variables. By going through a random walk, the nest, which is regarded as the central point of a cluster, is updated. The step length *s* is calculated by *s* = *u*/|*v*|^1/*β*^ and *β* ∈ [1,2], where *u* and *v* are drawn from normal distributions. That is, *u* ~ *N*(0, *σ*_*u*_^2^) and *v* ~ *N*(0, *σ*_*v*_^2^), where *σ*_*u*_ = [Γ(1+*β*)sin⁡(*πβ*/2)/Γ[(1+*β*)/2]*β*2^(*β*−1)/2^]^1/*β*^. This distribution follows the Levy distribution.

The goal of this clustering algorithm is to search for the best center to minimize the distance between the center of the cluster and its points. Our objective function is, thus, the same as (3) and its result is the degree of fitness. After calculating the degree of fitness, we use (4) to assign each point to a suitable cluster. A better degree of fitness represents a good quality cuckoo solution.

The best solution derived from the above equations is then nominated, and the new solution replaces the old. If no new solution is better than the old one, the current solution is retained as the best. The host bird cleans out the nest according to a given probability. The next iteration of the computation process then occurs to look for the next best solution. In each iteration, the best and hence the optimal solution to date is set as the clustering centroid. The centroid is represented as a paired tuple, cen(*i*, :), where *i* is the central point of the cluster. The tuple has the format of (*k*, *d*), where *k* is the *k*th cluster and *d* is the coordinates in (*x*, *y*, *z*,…etc.) or higher dimensions. For example, the locations of three clusters can be represented as [1, (3,4, 5), 2, (8,8, 9), 3, (5,6, 4)]. Each cuckoo represents a cluster with coordinates (*k*, *d*), and *d* is the central coordinate of the cuckoo. Each cuckoo is initially assigned a random *d* value. Subsequently, *d* is updated by iterative optimization. The progressive search for the best solution helps to avoid local optima in a manner similar to chromosome mutations in genetic algorithms. When the locations of the cuckoos are set in each round, the distances between the data points and the centers are measured by their Euclidean distance. The data points are reassigned to their nearest cluster by

(5) $$\begin{array}{c} \text{MIN}\left\{x-\text{cen}\right\}. \end{array}$$

The clusters are then reformed on the basis of the newly assigned data points. At the beginning, the averages of the data points are used as starting centroids by

(6) $$\begin{array}{c} \text{cen}=\frac{1}{N}\underset{x_{i}\in {\text{cluster}}_{i}}{\sum }x_{i},\;i=1,\ldots ,K. \end{array}$$

**Figure 2:**
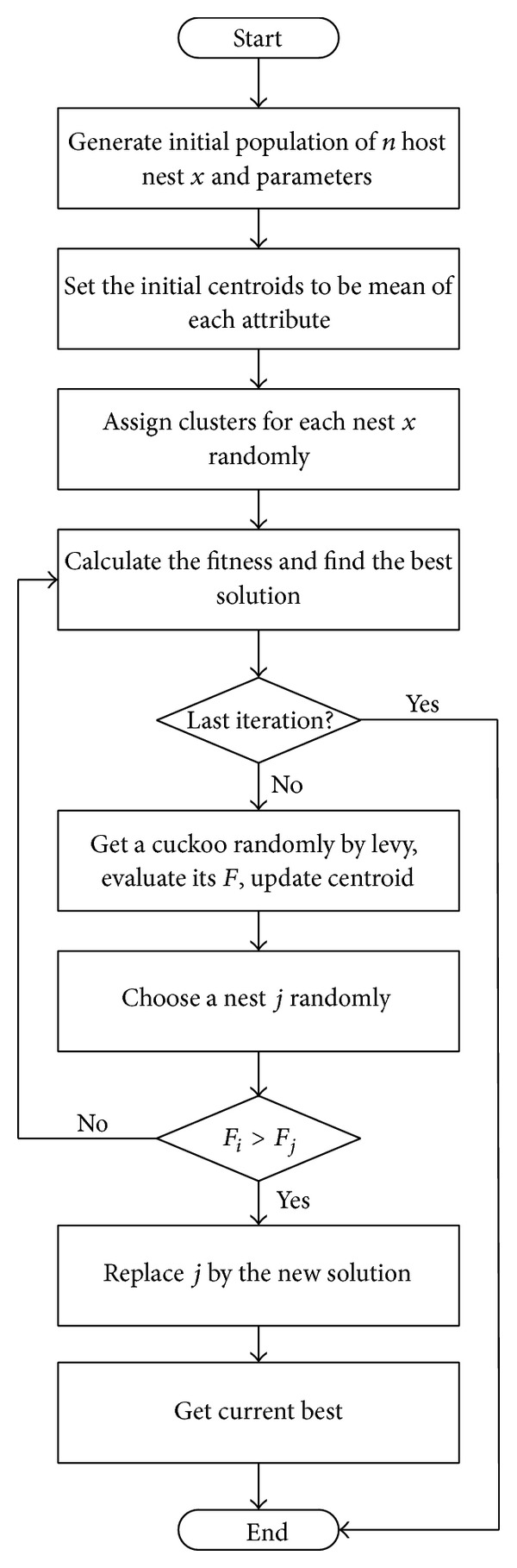
Workflow of C-Cuckoo algorithm.

In this way, the algorithm achieves better partitioning of clusters at the start to avoid the center points being too near or too far from each other, as would occur if they were assigned purely by random chance. As the algorithm runs, the clustering distribution is refined and changes to quality centroids are avoided by averaging the data. This is why (6) is only needed at the beginning to initialize the starting centroids. According to survival of the fittest, the partitioning process reaches a final optimum. The logic of the C-Cuckoo algorithm is shown as a flow chart in Figure 2.

### 2.2. Bat Clustering Algorithm (C-Bat)

Each bat has its own unique ID, position, and fitness attributes. However, in the bat algorithm, each bat is assigned the same loudness, pulse frequency, and wave length. The position of a bat is represented by a solution *x*. Its location is determined by the values of *D* dimensions. As for the C-Cuckoo algorithm, the solution uses a (*N*, *K* ⊗ *D*) matrix, the second term identifying the location of the bat.

The initiation step is similar to that employed for the C-Cuckoo algorithm. However, the bats have an additional feature: each bat has a velocity *v*_*i*_, which is similar to particle swarm optimization. The bat's position is partly determined by its velocity. At first, the bats are randomly distributed. After initialization, the bats move to a better place according to (8). A random number is then produced: if it is larger than the current bat rate, the algorithm selects a solution from those calculated and generates a local solution. The centroids are the averages of the nearby data points. The distances are then minimized according to the direction of the optimization goal. The objective functions are identical to (5) and (6) above. The convergence process then starts to iterate based on the following formula:

(7) $$\begin{array}{c} \beta =Q\times \text{rand}\left(\right), \end{array}$$

where rand() is a random value generator and the random values are distributed over [0,1]. Consider

(8) $$\begin{array}{c} f_{i}=f_{\min}+\left(f_{\max}-f_{\min}\right)\times \beta ,\\ v_{i}^{t}=v_{i}^{t-1}+\left(x_{i}^{t-1}-x_{*}\right)\times f_{i},\\ x_{i}^{t}=x_{i}^{t-1}+v_{i}^{t}. \end{array}$$

The positions of the bats are then updated. *f* represents the frequency of echolocation. When the frequency equals the sum of the minimum frequency and the difference between the maximum and minimum frequencies, the speed of the bat is updated. The new speed is set to the previous speed plus the product of the previous frequency and the difference between the current position and the previous position. A variable called the pulse rate is also used in the algorithm. When the pulse rate is exceeded, the following formula is updated:

(9) $$\begin{array}{c} x_{i}^{t}=x_{*}+0.01\times \text{rand}\left(\right). \end{array}$$

**Figure 3:**
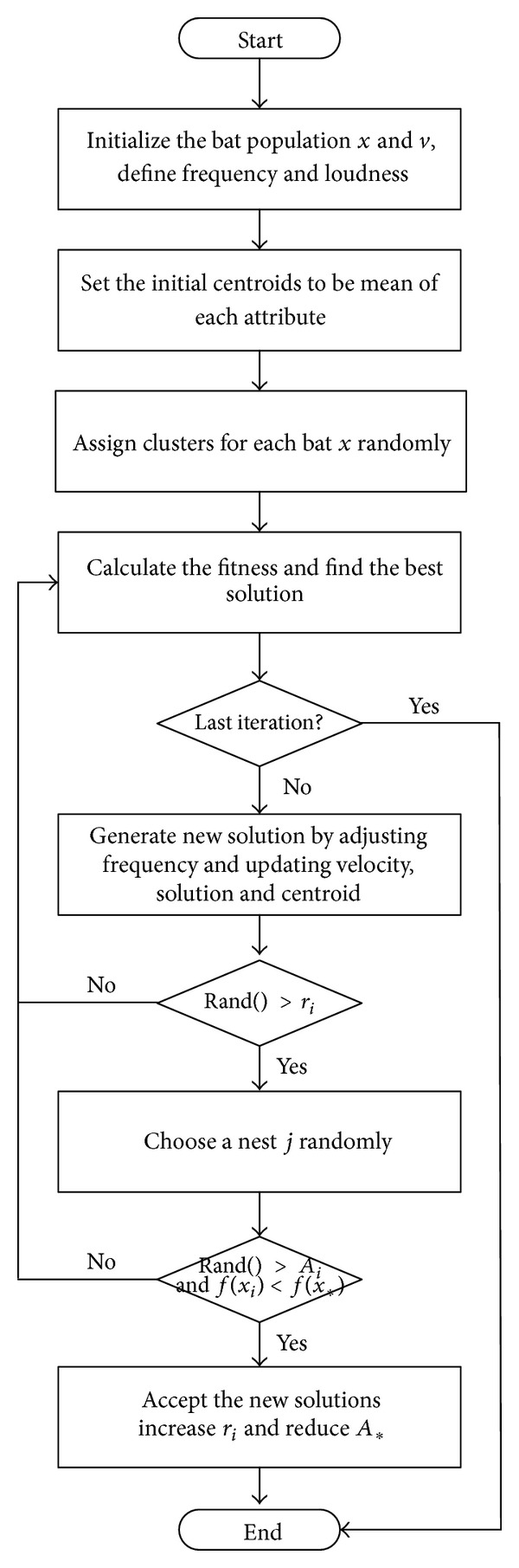
Workflow of C-Bat algorithm.

Equation (9) serves as the updating function, where *x*_∗_ is taken as the best solution. It is also used to represent the best position for the bat to move towards. If the loudness value is not high enough and the new solution is better than the old one, the better one becomes the solution. A fitness function the same as that employed for the C-Cuckoo algorithm is then applied by checking whether echolocation is loud enough. The logic of the C-Bat algorithm is shown as a flow chart in Figure 3.

## 3. Experiments

There are two sets of experiments; one is focused in evaluating the performance of algorithms using a series of multivariate real-life datasets. The other is to test out the efficacy of the algorithms in image segmentation. The purpose is to validate the new algorithms with respect to their clustering quality. The first test is about how the new algorithms work with general-purpose datasets with different number of attributes and instances. Their performances in particular are evaluated in details. The latter test is to observe how well these algorithms will work in the domain of machine vision. The setups of the experiments and their results are discussed as follow.

### 3.1. Experiment Set-Up

The new nature-inspired clustering algorithms (C-ACO, C-Bat, C-Cuckoo, and C-Firefly) proposed here are experimented over six datasets which are available for download from UCI data repository (http://archive.ics.uci.edu/ml). The clustering results of the new hybrid clustering algorithms are compared with those of original K-means which serves as a benchmarking reference. The computer simulation environment is implemented in MATLAB software and the algorithms are coded in MATLAB programs. The hardware platform is a MacBook Pro computer configured with a 2.3 GHz CPU and 4 GB RAM. In each trail run, each of the testing datasets is run repeatedly ten times for measuring up the average CPU time consumption, as well as obtaining the average values of the top objective fitness. The datasets used are collected from real-life applications, namely, Iris, Wine, Haberman's survival, Libras, and Synthetic. Synthetic is a dataset of control chart time-series which are artificially generated clustering data point values in random. The information regarding the testing datasets is shown in Table 4.

The full length of dataset is used for training—in clustering, building clusters are referred to until perfection is attained using the full set of data. Performance of the clustering is evaluated in terms of cluster integrity which is reflected by the intra- and intersimilarities of data points within and across different clusters, the average sum of CPU time consumption per iteration during the clustering operation, and the number of loops taken for all the clusters to get converged. The criterion for convergence which decides when the looping of evolution stops is the fraction of the minimum distance between the initial cluster centers that takes on a numeric value between [0, 1]. For example, if the criterion is initialized with 0.01, the looping halts when a complete iteration does not move any of the cluster centers by a distance of more than 1% of the smallest distance between any of the initial cluster centers.

The quality of the final outcome of clustering is measured by the integrity of each of the clusters, which in turn is represented by the final fitness value of the objective function. The resultant fitness value of the objective function is driven by how much each variable contributes towards the final goal which is being optimized in the process. From the perspective of clustering the goal is finding a suitable set of centroids as guided by the metaheuristic of the nature-inspired algorithm. The metaheuristic will always insist that the relocation of centroids in each step is progressive aiming at exploring for the optimum grouping. The end result of the ideal group should lead to having the data points within each cluster closest to their centroid. Iteratively, the search for the optimum grouping proceeds. During the search the *k* centroids relocate in the search space step-by-step according to the swarming pattern of the nature-inspired optimization algorithm until no more improvement is observed. It stops when there is no further relocation that will offer a better result. To be precise, no other new relocation of centroids seems to provide better integrity of the clusters. The algorithm is geared at minimizing the intrasimilarity of each cluster by an objective function. In this case, it is a squared error function so any slight difference will be enlarged by the square function. Equation (10) defines such objective function. Consider

(10) $$\begin{array}{c} V=\overset{K}{\underset{j=1}{\sum }}\;\overset{N}{\underset{i=1}{\sum }}{||x_{i,j}-{\text{cen}}_{j}||}^{2}. \end{array}$$

In the experiments, each dataset is run ten times to test and obtain the average CPU time and is also run ten times to test the objective function values/best fitness value. The parameters are set as reported in Tables 5 and 6.

### 3.2. Testing the New Clustering Algorithms with General-Purpose Multivariate Datasets

**Figure 4:**
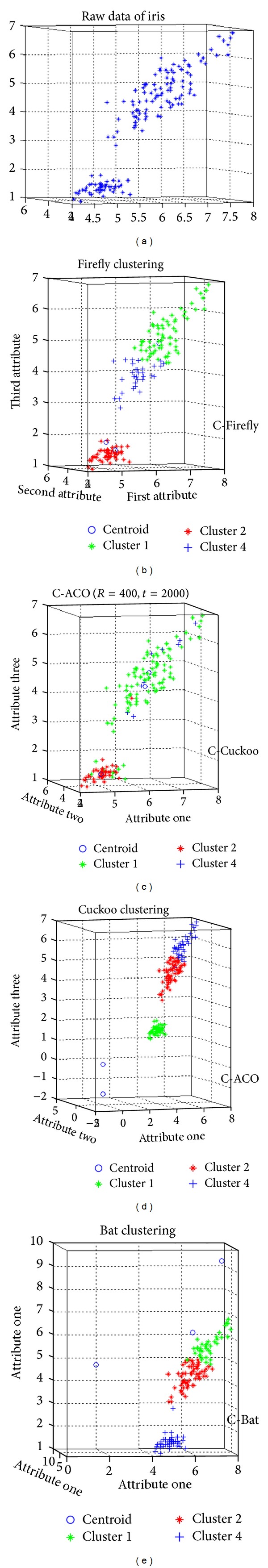
Snapshots of clustering operations.

The five diagrams in Figure 4 below present snapshots of the experimental run for the Iris dataset. The original data points are shown in the topmost plot Figure 4(a), and the data points in different colors obtained by the new clustering algorithms are shown in Figures 4(b), 4(c), 4(d), and 4(e) by C-Firefly, C-Cuckoo, C-ACO, and C-Bat, respectively.

The quantitative experimental results are shown in Tables 7, 8, 9, 10, and 11. To make observation easier, the best result across the four algorithms under test (in each column) is highlighted with a double asterisks. It is apparent to observe that the C-Cuckoo and C-Bat algorithms achieve a lot better of objective fitness value than do the C-ACO and C-Firefly algorithms. Overall, the four nature-inspired clustering algorithms execute in less time and succeed in achieving higher accuracy in clustering than the plain K-means. High accuracy is referred to high cluster integrity where the data points in a cluster are close to their centroid. This observation tallies with our proposition that K-means enhanced by nature-inspired optimization algorithms speeds up the searching time for the good centroids for good clustering integrity. This enhancement is important because for all the partitioning-based clustering methods potentially they can be enhanced by nature-inspired algorithms in the similar way—the end result is expected in search process acceleration and local optima avoidance.

Our detailed performance evaluation tests include computing the final objective function fitness values and the CPU time consumption for clustering the data in the general-purpose UCI datasets in Table 4. The objective function fitness value is computed using (10) which represents the overall cluster integrity, and CPU time consumption is timed as how long it is necessary for the clustering algorithm to converge from beginning to end. Given the datasets are of fixed volume, CPU time consumption is related directly to the speed of the clustering operation.

Tables 7 to 11 clearly show that the C-Cuckoo and C-Bat algorithms both yield better objective values than the C-ACO and C-Firefly algorithms. The study reported in [1] has already shown that the C-ACO and C-Firefly algorithms perform more quickly and accurately than a traditional K-means specification. Our evaluation results provide further evidence confirming this phenomenon: nature-inspired algorithms do indeed accelerate the process of finding globally optimum centroids in clustering, and partitioning clustering methods can be combined with nature-inspired algorithms to speed up the clustering process and avoid local optima. Furthermore, our results show that two new hybrid clustering algorithms, the C-Cuckoo and C-Bat specifications, are more efficient and accurate than the others we test.

The next experiment is undertaken to measure the average computation time required per iteration in the clustering process. Only the Iris dataset is used here, as it is one of the datasets in the UCI repository most commonly used for testing time spent per iteration for clustering with nature-inspired algorithms.

**Figure 5:**
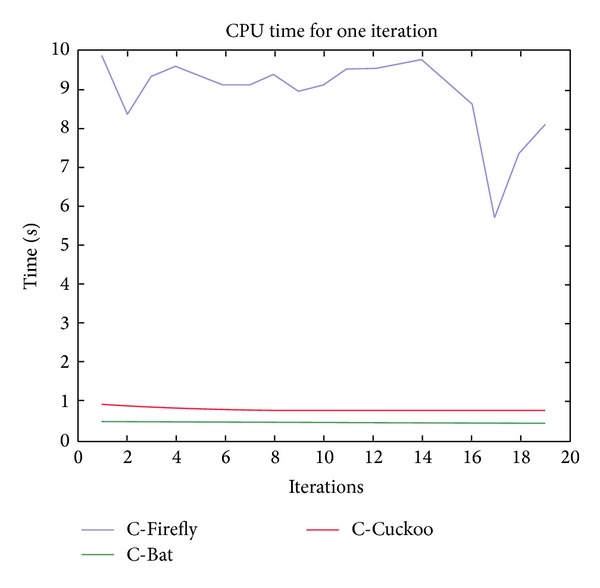
Computation time (in secs.) for C-Firefly, C-Cuckoo, and C-Bat algorithms.

**Figure 6:**
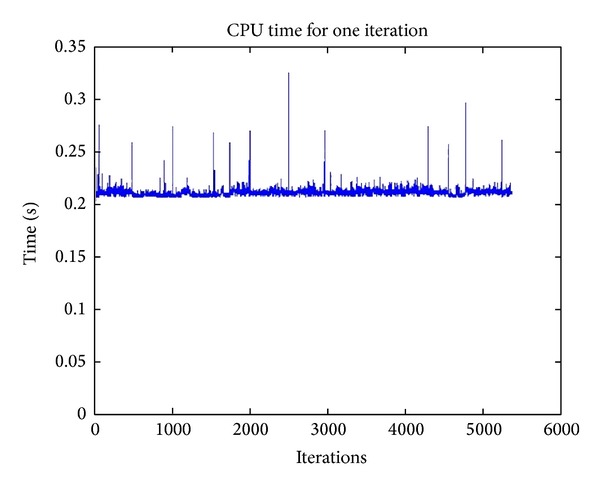
Computation time (in secs.) for C-ACO algorithm.

From Figures 5 and 6, it can be seen that all four algorithms scale quite well—as the number of iterations increases, the computation time taken remains flat. In particular, C-ACO is very fast. It takes only a fraction of a second to execute each iteration of the clustering process. C-Firefly takes about 8 to 10 seconds. C-Cuckoo and C-Bat are relatively fast, taking less than a second per iteration for code execution. The CPU times taken for each algorithm are reported in Table 12. The figures are averaged, and the table shows the net CPU time taken per iteration.

**Figure 7:**
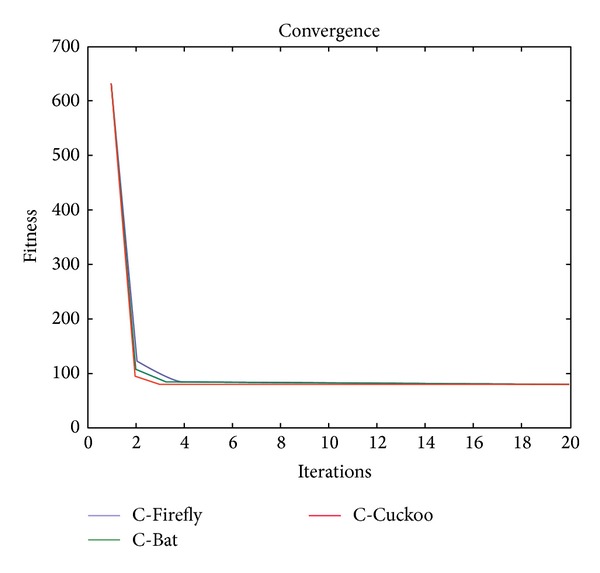
Number of iterations required for C-Firefly, C-Cuckoo, and C-Bat algorithms to converge.

**Figure 8:**
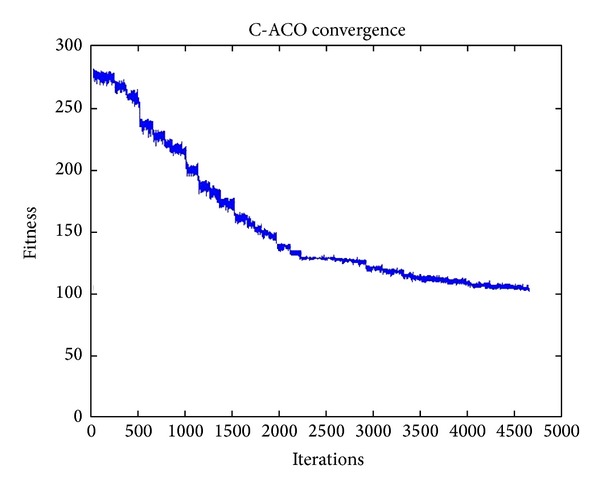
Number of iterations required for C-ACO to converge.

The following graphs in Figures 7 and 8 show the number of iterations required for the clustering algorithms to converge according to the given threshold criterion.

As shown in Figure 7, the C-Firefly, C-Cuckoo, and C-Bat algorithms take about two or three iterations to achieve convergence, which is extremely fast. In contrast, C-ACO in Figure 8 takes many rounds to converge, 4681 iterations to be exact. For the other three algorithms, the best objective function value is reached at 78.94. C-ACO goes no lower than 101 and remains there even if the number of iterations increases to a large value.

Therefore, we may conclude that the C-Firefly, C-Cuckoo, and C-Bat algorithms are suitable for static data. C-Firefly compares data *O*(*n* ^2^) times in each iteration, so it takes a lot of time to converge. The traditional K-means algorithm converges easily to a local optimum, so the result of the objective function is worse than for the others. C-Bat has the ability to adjust itself in every iteration, and because it only changes location once, at the end of an iteration, it is very fast. Because C-Cuckoo retains better solutions and discards worse solutions, working like a PSO-clustering algorithm, it also performs well in providing objective function values.

Although C-Bat, C-Cuckoo, and C-Firefly may need more time for each iteration, they are good optimization algorithms. They can find the optimal solution relatively quickly overall (because they converge very fast). However, the ants acting as the searching agents in the C-ACO algorithm make only a small move in each iteration. Many comparisons are thus required to find the best solution. In sum, C-ACO may be suitable for applications in which incremental optimization is desired and very little time is needed for each step, but it may take many steps to reach the optimal goal.

The next set of experiments tests the quality of clustering in terms of accuracy (measured as 100% minus the percentage of instances overlapping in wrong clusters) and standard deviation. Standard deviation is related to how much variation from the average (mean) is caused by clustered data. The misclustered data derived in the experiments can be seen in Figure 3. Most of the results are satisfactory. The standard deviations indicate that the data points tend to be very close to the mean. The mathematical definition is simply

(11) $$\begin{array}{c} \sigma =\sqrt{\frac{1}{N}\left[{\left(x_{1}-u\right)}^{2}+{\left(x_{2}-u\right)}^{2}+\cdots +{\left(x_{N}-u\right)}^{2}\right]}, \end{array}$$

where *u* = (1/*N*)(*x*_1_ + ⋯+*x*_*N*_). Again, the most widely employed dataset, the iris dataset, is used for this set of experiments.

Table 13 shows that the results obtained using the C-Bat algorithm are the best in the iris dataset, whereas those derived with the C-Cuckoo algorithm are the best in the wine dataset. In the Haberman data, all five algorithms are almost equally accurate, though the C-ACO algorithm is slightly more precise.

Table 14 shows that the C-Firefly algorithm has the minimum deviation within clusters, whereas the original K-means algorithm deviates to the greatest extent.

### 3.3. Testing the New Clustering Algorithms in Image Segmentation

In this set of experiment, the new hybrid clustering algorithms are put under test of image segmentation task. Pixel color oriented image segmentation is the core of image analysis which finds its applications in many areas of image interpretation, pattern identification/recognition, and robotic vision. Some popular applications [13] include but are not limited to geographical information remote sensing, medical microscopy, content-based audio/visual media retrieval, factory automation, and unmanned vehicle navigation, just to name a few.

In practical scientific and industrial applications, the quality and accuracy of image segmentation are very important which depend on the underlying data clustering algorithms. A common choice of unsupervised clustering algorithm is K-means in image segmentation based on color. The regions of the image depending on the color features are grouped into a certain set of segments by measuring the intercluster distance and intracluster distance between each image pixel and the centroid within the cluster. The clustering process is exactly the same as that used in the previous experiment on UCI datasets, except that the images under test in this experiment are larger in amount. An 8 MB high-resolution photo like those used in the experiment here has typically 5184 × 3456 pixels. In addition to spatial information, *x*- and *y*-axises of the pixel position in the image, each pixel is triplet of red, green, and blue information, ranging from 0 in darkness to 255 being the strongest in intensity. It is well-known that every pixel of an image is made up by mixing the 256 independent intensity levels of red, green, and blue light. Each data point of the 17,915,904 pixels is a five-dimensional matrix comprised of the pixel location information and RGB information, [*x*, *y*, *R*, *G*, *B*] where 0 ≤ *x* ≤ 5184, 0 ≤ *y* ≤ 3456, and 0 ≤ *R*, *G*, *B* ≤ 255. The hybrid clustering algorithms are extended from K-means in the same way as described in Section 2. The required image data can be technically extracted by using MATLAB functions* imread*(*filename*) that creates a three-dimensional matrix and* impixel*() that returns the values of the RGB triplet for the pixel.

The experiment is run over four images whose pixels are to be clustered using different preset numbers of *k* = 3 and *k* = 4. The four images are shots of sceneries, namely, Tower Bridge (TB), Cambridge University (CU), Le Mont-Saint-Michel (MSM), and Château de Chenonceau (CDC). They have similar image composition and identical size. Some particular features that are subtly contained in the images are used for testing the efficacy of the clustering algorithms like those as follows.

- TB, thickness of clouds in the sky, the shaded part of the tower bridge.
- CU, depths of perspectives along the cupula and college building, details on the lawn.
- MSM, details of the fortress wall and small windows on the chapel.
- CDC, reflection of the Château over the water.

**Figure 9:**
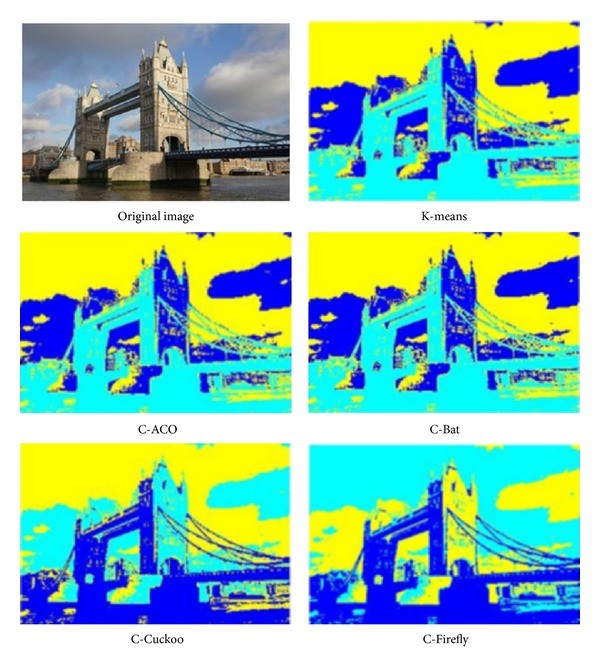
Results of image segmentation by using different nature-inspired clustering algorithms, on a photo called “Tower Bridge.”

**Figure 10:**
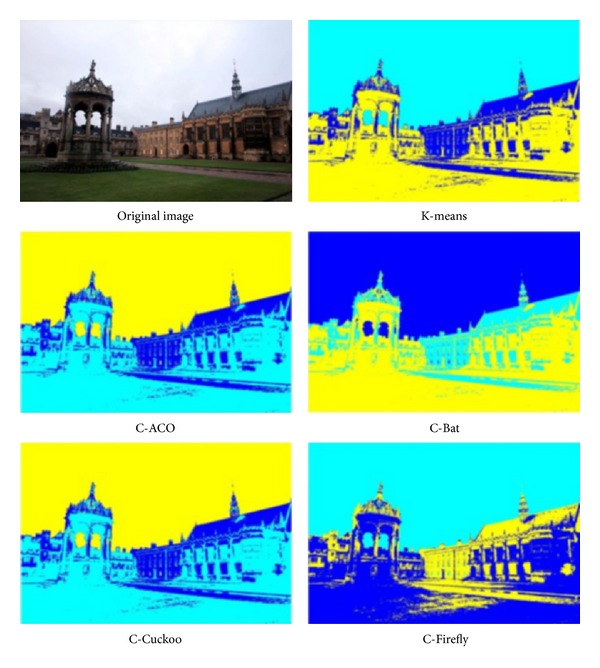
Results of image segmentation by using different nature-inspired clustering algorithms, on a photo called “Cambridge University.”

**Figure 11:**
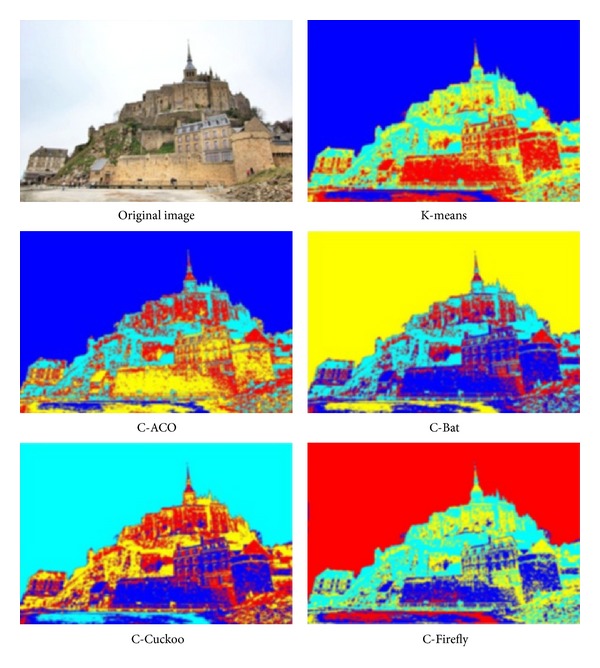
Results of image segmentation by using different nature-inspired clustering algorithms, on a photo called “Le Mont-Saint-Michel.”

**Figure 12:**
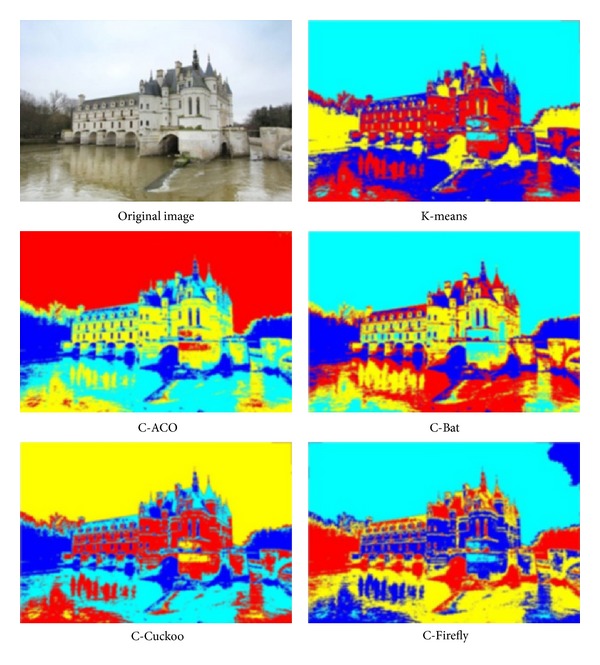
Results of image segmentation by using different nature-inspired clustering algorithms, on a photo called “Château de Chenonceau.”

The performance is again measured by intersimilarity and intrasimilarity across and within the same cluster, as well as time taken in seconds in processing a whole image. The performance results are tabulated in Table 15. The winning performance result by one of the four hybrid clustering algorithms or K-means is marked with a triple asterisks as distinction. The original images under test and the segmented images by the various clustering algorithms are shown in Figures 9, 10, 11, and 12, respectively for TB, CU, MSM, and CDC.

The performance is again measured by intersimilarity and intrasimilarity across and within the same cluster, as well as time taken in seconds in processing a whole image. The performance results are tabulated in Table 15. The winning performance result by one of the four hybrid clustering algorithms or K-means is marked with a triple asterisks as distinction.

In all the tests, K-means seems to take the shortest time, probably because it stops early in local optima. This is evident by the fact that none of the results by K-means score the best in either intercluster distance or intracluster distance. In the experiment of TB, C-ACO scores the widest intercluster length, and C-Firefly has the tightest intracluster distance. As a result, visually C-ACO produces slightly more details on the cloud at the top right corner. C-Firefly seems to produce the most details on the sun-facing side of the tower as well as the shaded side of the tower. C-Firefly again scores the best in intracluster distance in CU and CDC. Again, in CU, C-Firefly offers the most details on the lawn; the copula likewise has most details and reproduces seemingly most accurately on the college façade by C-Firefly. Interestingly, C-Cuckoo has the longest inter,cluster distance in MSM and CDC. In MSM C-Cuckoo gives the most structural outline of shades and colors on the wall of the chapel, while C-Firefly produces most details on the fortress wall. In CDC, C-Cuckoo and C-Firefly manage to produce the relatively best reflection images over the water, by visual inspect.

The overall results of the experiments described in this paper show that two of the new clustering algorithms observed here, the C-Cuckoo and C-Bat algorithms, which have never been tested by other researchers, are more efficient and accurate than the C-ACO and C-Firefly specifications. This represents a significant contribution to existing knowledge, because it sheds light on the encouraging possibility that optimization techniques derived from nature can be used to improve K-means clustering; we hope that this lays the foundation for more sophisticated bio-inspired optimization methods to be integrated with existing clustering algorithms.

The characteristics of each of the four bioinspired clustering algorithms are listed in the Appendix by way of summary. It is hoped that researchers will find them useful as a source of inspiration for developing better algorithms in future. As the phrase “meta-heuristics” suggests, these bioinspired optimization heuristics come in abstract and general forms. There is ample potential to extend, modify, and even build hybrids of them with other heuristic functions to suit different applications.

## 4. Conclusion

K-Means clustering algorithms, a classical class of partition-based algorithms used for merging similar data into clusters, are known to have the limitation of getting stuck in local optima. As a matter of intellectual curiosity in computer science, how best to cluster data such that the integrity of the clusters is maximized, has always been a challenging research question. The ideal solution is to find an optimal clustering arrangement which is globally best—so that no other possible combinations of data clustering exist that are better than the global one. One way of achieving this is to try all the possible combinations by brute-force which could be computational intractable. Alternatively, nature-inspired optimization algorithms, which recently rise as a popular research topic, are extended to work with K-means in guiding the convergence of disparate data points and to steer them towards global optima, stochastically instead of deterministically. These two research directions of metaheuristic optimization and data mining do fit like hand and glove. Constrained by the inherent limitation of K-means design and the merits of nature-inspired optimization algorithms, it is feasible to combine them letting them complement and function together. This paper evaluates four hybrid types of clustering algorithms developed by integrating nature-inspired optimization algorithms into K-means. The results produced from the experiments clearly validate the new algorithms possess a performance enhancement, apparently for the C-Bat and C-Cuckoo. The extended versions of clustering algorithms enhanced by nature-inspired optimization methods perform better than their original versions, in two sets of experimental datasets—general purpose and image segmentation. Experiments are conducted to validate the benefits of the proposed approach.

## Figures and Tables

**Table 1:** Parameters used for C-Firefly.

*N*	Population of fireflies
*M*	Number of pseudo time steps
*K*	Number of groups or clusters
*S*	Number of samples
*D*	Number of attributes
*w*(*s*, *k*)	The element in matrix [*s*, *k*] where *s* ∈ *S*, *k* ∈ *K*
	$w_{s,k}=\{\begin{array}{cc} 1, & x_{s}\in {\text{cluster}}_{k}\\ 0, & x_{s}\notin {\text{cluster}}_{k} \end{array}$
*x*(*i*, *j*)	The best solution in matrix [*i*, *j*] where *i* ∈ *N*, *j* ∈ *K* ⊗ *D*
Cen(*k*, *d*)	The centroid in matrix [*k*, *d*] where *k* ∈ *K*, *d* ∈ *K* ⊗ *D*
Clmat(*n*, *s*)	The classification matrix [*n*, *s*] where *n* ∈ *N*, *s* ∈ *S*

Table 1 consists of the parameters for the C-Firefly algorithm. *X* is a composite matrix of size [*N*, (*K* ⊗ *D*)], where *x* ∈ *X* since *X* has a maximum of *K* centroids and each centroid is represented by a maximum of *D* dimensions by the attributes.

**Table 2:** Parameters used for C-Cuckoo.

*N*	Population of cuckoos
*M*	Number of pseudo time steps
*Pa*	Discovery rate of alien eggs
Tol	Tolerance
*K*	Number of groups (clusters)
*S*	Number of samples
*D*	Number of attributes
*w*(*s*, *k*)	The element in matrix [*s*, *k*] where *s* ∈ *S*, *k* ∈ *K*
	$w_{s,k}=\{\begin{array}{cc} 1, & x_{s}\in {\text{cluster}}_{k}\\ 0, & x_{s}\notin {\text{cluster}}_{k} \end{array}$
*x*(*i*, *j*)	The best solution in matrix [*i*, *j*] where *i* ∈ *N*, *j* ∈ *K* ⊗ *D*
Cen(*k*, *d*)	The centroid in matrix [*k*, *d*] where *k* ∈ *K*, *d* ∈ *K* ⊗ *D*
Clmat(*n*, *s*)	The classification matrix [*n*, *s*] where *n* ∈ *N*, *s* ∈ *S*

*Pa* and Tol are conditional variables used for controlling execution of the cuckoo optimization algorithm [9].

**Table 3:** Parameters used for C-Bat.

*Q*	Frequency
*V*	Velocity
*R*	Pulse rate
*A*	Loudness
*⋯*	All other parameters are the same as those in Table 1

**Table 4:** Testing dataset information.

Dataset	Instances	Attributes	Clusters
Iris	150	4	3
Wine	178	13	3
Haberman' s survival	306	3	2
Libras	360	91	15
Synthetic	600	60	6

**Table 5:** Parameters set for C-Firefly and C-Cuckoo.

C-Firefly	C-Cuckoo
*α* (randomness): 0.2	*Pa* (discovery rate): 0.25
*γ* (absorption): 1.0	Tol (tolerance): 1.0*e* ^−5^
Number of clusters: 4	*β* (Levy): $\frac{3}{2}$
Max number of iterations: 20	*ε* (Levy): $\sigma_{u}={\left[\frac{\Gamma (1+\beta )\sin(\pi \beta /2)}{\Gamma [\left(1+\beta \right)/2]\beta 2^{(\beta -1)/2}}\right]}^{1/\beta }$
Population: 400	Number of clusters: 4
*β* = 0	Max number of iterations: 20
*β*_0_ = 0	Population: 400

**Table 6:** Parameters set for C-Bat and C-ACO.

C-Bat	C-ACO
*A* (loudness): 0.5	Population: 400
*R *(pulse rate): 0.5	*q* (threshold): 0.9
Number of clusters: 4	Number of clusters: 4
Max number of iterations: 20	*rho* (evaporation rate): 0.1
*Q*_min⁡_ (frequency min): 0	Number of clusters: 4
*Q*_max⁡_ (frequency max): 0.2	Max number of iterations: 20
Population: 400	

**Table 7:** Testing objective function fitness and CPU time consumption on IRIS.

Objective function value			
	Best	Worst	Average
K-means	78.9451	152.3687	127.8941
C-ACO	139.3081	150.9815	144.79
C-Firefly	78.9408**	109.4036	89.88241
C-Cuckoo	78.9408**	78.9408**	78.9408**
C-Bat	78.9408**	81.2655	79.46626

CPU time (second)			
	Best	Worst	Average

K-means	51.291414	51.673431	51.444755
C-ACO	440.7980	443.9105	443.0668
C-Firefly	142.899517	177.743484	167.2426438
C-Cuckoo	15.499098	15.798136	15.7319299
C-Bat	8.6212**	8.8354**	8.75495**

**Table 8:** Testing objective function fitness and CPU time consumption on wine.

Objective function value			
	Best	Worst	Average
K-means	2.3707*e* + 006	2.3707*e* + 006	2.3707*e* + 006
C-ACO	2.3707*e* + 006	2.3707*e* + 006	2.3707*e* + 006
C-Firefly	2.3707*e* + 006	2.3707*e* + 006	2.3707*e* + 006
C-Cuckoo	2.3707*e* + 006	2.3707*e* + 006	2.3707*e* + 006
C-Bat	2.3707*e* + 006	2.3707*e* + 006	2.3707*e* + 006

CPU time (second)			
	Best	Worst	Average

K-means	73.793875	77.226464	75.7386
C-ACO	623.3123	695.2312	653.2154
C-Firefly	260.725002	278.206271	267.8236183
C-Cuckoo	18.582329	19.137426	18.7511367
C-Bat	10.3032**	10.8225**	10.42148**

**Table 9:** Testing objective function fitness and CPU time consumption on Libras.

Objective function value			
	Best	Worst	Average
K-means	822.8381	899.2441	842.3452
C-ACO	1.1361*e* + 003	1.6345*e* + 003	1.3981*e* + 003
C-Firefly	743.3432	892.0506	777.1993
C-Cuckoo	707.5916**	819.1392**	763.1102**
C-Bat	745.8008	918.2488	841.71931

CPU time (second)			
	Best	Worst	Average

K-means	1332.059811	1392.3113	1362.0344
C-ACO	1.0142*e* + 004	1.3245*e* + 004	1.1195*e* + 004
C-Firefly	260.725002	278.206271	267.8236183
C-Cuckoo	168.502947	169.849548	169.17942
C-Bat	10.3032**	10.8225**	10.42148**

**Table 10:** Testing objective function fitness and CPU time consumption on Haberman.

Objective function value			
	Best	Worst	Average
K-means	30507.0207	31321.2134	30912.2141
C-ACO	2888.4833**	3051.1412**	2933.1641**
C-Firefly	30507.2735	31567.7231	30822.3426
C-Cuckoo	30507.0207	30574.2412	30591.1234
C-Bat	30507.8928	31455.1241	30712.8321

CPU time (second)			
	Best	Worst	Average

K-means	170.0535	174.123152	172.743
C-ACO	825.2858	856.3724	831.223
C-Firefly	1257.6772	1289.3124	1266.4123
C-Cuckoo	25.1611	26.2312	25.4431
C-Bat	14.5597**	14.9087**	14.6722**

**Table 11:** Testing objective function fitness and CPU time consumption on Synthetic.

Objective function value			
	Best	Worst	Average
K-means	9.8221*e* + 005	1.0451*e* + 006	9.9632*e* + 005
C-ACO	43847.7264**	5.4264.3234**	47321.9682**
C-Firefly	9.4509*e* + 005	9.8221*e* + 005	9.6622*e* + 005
C-Cuckoo	9.4499*e* + 005	9.7800*e* + 005	9.5721*e* + 005
C-Bat	9.5232*e* + 005	9.8525*e* + 005	9.6150*e* + 005

CPU time (second)			
	Best	Worst	Average

K-means	1579.2311	1663.2234	1617.305490
C-ACO	4354.9328	4725.4525	4556.2073
C-Firefly	51092.57336	51404.66323	51232.55324
C-Cuckoo	138.204069	151.547294	141.9006882
C-Bat	77.8049**	80.8928**	79.63274**

**Table 12:** Comparison of algorithms with respect to CPU time taken per iteration.

CPU time per iteration			

Iris			
	Best	Worst	Average

C-ACO	0.2148**	0.2625**	0.2260**
C-Firefly	8.1591	9.8726	8.5261
C-Cuckoo	0.7824	0.8812	0.8035
C-Bat	0.4340	0.5066	0.4448

Wine			
	Best	Worst	Average

C-ACO	0.3362*	0.5362*	0.4271*
C-Firefly	10.6828	16.4298	12.5449
C-Cuckoo	0.937	1.0074	0.95102
C-Bat	0.5231	0.5576	0.52809

Haberman			
	Best	Worst	Average

C-ACO	0.4156*	0.4551*	0.4243*
C-Firefly	64.3562	67.9928	66.1953
C-Cuckoo	1.2376	1.3037	1.2581
C-Bat	0.4340	0.5066	0.4448

**Table 13:** Accuracy of clustering in the three datasets using different clustering algorithms.

Accuracy of clustering algorithm			
Name	Iris	Wine	Haberman
K-means	0.8666	0.7225**	0.522
C-Firefly	0.7866	0.7225**	0.529
C-ACO	0.68	0.5505	0.562**
C-Cuckoo	0.89	0.7225**	0.526
C-Bat	0.92**	0.6966	0.473

**Table 14:** Standard deviation measures for different algorithms in different datasets.

The standard deviation of each algorithm			

Iris			
Algorithm	Cluster1	Cluster2	Cluster3

K-means	1.8476**	1.7277	1.9847
C-ACO	1.9185	1.809	1.9742
C-Firefly	1.8476**	1.6269**	1.9136**
C-Cuckoo	1.8476**	1.7345	1.982
C-Bat	1.8476**	1.7311	1.9741

Wine			
Algorithm	Cluster1	Cluster2	Cluster3

K-means	319.8828	194.4313	123.3868
C-ACO	296.271**	155.1661**	154.7441
C-Firefly	319.8828	194.4315	123.3868
C-Cuckoo	319.8828	194.4315	123.3836**
C-Bat	318.5658	200.5721	130.7622

Haberman			
Algorithm	Cluster1	Cluster2	

K-means	28.1977	25.1797	
C-ACO	28.0195**	25.4671	
C-Firefly	28.0842	25.1372**	
C-Cuckoo	28.1977	25.1797	
C-Bat	28.1627	25.193	

**Table 15:** Performance of clustering results of image segmentation using different clustering Aalgorithms.

Algorithms	Intercluster distance	Intracluster distance	CPU time (s)
Tower Bridge, *k* = 3			
K-means	6.5932*e* + 4	1.1154*e* + 8	24.5465***
C-ACO	6.7741*e* + 4***	1.0561*e* + 8	351.9310
C-Bat	6.5932*e* + 4	1.0561*e* + 8	39.3933
C-Cuckoo	6.7412*e* + 4	1.0561*e* + 8	39.7490
C-Firefly	3.5565*e* + 4	1.0407*e* + 8***	150.1532

Cambridge University, *k* = 3			
K-means	1.7154*e* + 5	4.8854*e* + 8	166.7963∗∗∗
C-ACO	1.9773*e* + 5	4.8926*e* + 8	343.1299
C-Bat	2.0971*e* + 5***	4.9134*e* + 8	386.0401
C-Cuckoo	1.9967*e* + 5	4.8915*e* + 8	380.7575
C-Firefly	0.0046*e* + 5	3.4682*e* + 8***	1477.0564

Le Mont-Saint-Michel, *k* = 4			
K-means	1.8142*e* + 5	2.5541*e* + 8	160.2645∗∗∗
C-ACO	1.7534*e* + 5	2.5273*e* + 8	223.5515
C-Bat	1.8125*e* + 5	2.5793*e* + 8	250.0147
C-Cuckoo	2.0102*e* + 5***	2.4998*e* + 8***	250.7541
C-Firefly	0.4564*e* + 5	2.5273*e* + 8	941.8844

Château de Chenonceau, *k* = 4			
K-means	1.7651*e* + 5	2.8993*e* + 8	148.6572∗∗∗
C-ACO	2.0091*e* + 5	2.5037*e* + 8	226.8645
C-Bat	1.8708*e* + 5	2.8424*e* + 8	251.7009
C-Cuckoo	2.0756*e* + 5***	2.4884*e* + 8	251.8786
C-Firefly	0.4310*e* + 5	1.5130*e* + 8***	982.2208

## Appendix

*C-Firefly*. (1) In this algorithm, using light intensity as the objective function value, each firefly *x* is represented by its location.

(2) Every firefly needs *O*(*n* ^2^) comparisons, and each one will move towards the brighter one. Therefore, it takes more time for each iteration, but convergence can be reached in very few iterations.

(3) The attractiveness varies according to the inverse square law (*r*) = *β*_0_ *e* ^−*γ* · *r*^2^^, the *γ* is the absorb coefficient.

*C-ACO. *(1) This algorithm uses a pheromone matrix as the communication channel.

(2) The pheromone matrix leads the ant to choose the path.

(3) Ants construct the solution step-by-step, and the decision variables of associated mathematical objective function are discrete.

*C-Cuckoo. *(1) The nests represent the population.

(2) The cuckoo moves using Levy flight, which is a Markov chain in which the next location depends solely on the current location.

(3) This algorithm has the ability to discover foreign eggs and abandon them. This means that the algorithm can avoid local optima.

(4) In each iteration, the worst solution will be replaced by the better one. If there is no worse solution, the better one will be retained for the next iteration.

(5) Every cuckoo only cares about its nest. It is not necessary to communicate.

*C-Bat. *(1) Each bat has data on velocity and location. This is similar to PSO-clustering.

(2) Velocity is determined by frequency, loudness, and pulse rate.

(3) The solution can be adjusted by frequency, loudness, and pulse rate.

(4) In each iteration, the bat only updates its location once. Therefore, the algorithm runs very quickly.

(5) The bat has the ability to adjust itself. It can sense the surrounding environment.

Similarities among these algorithms.

1. They all use attribute means to determine the initial centroid.
2. Optimization algorithms are the key factors in accelerating clustering and avoiding local optima.
